# Marked Increase in PROP Taste Responsiveness Following Oral Supplementation with Selected Salivary Proteins or Their Related Free Amino Acids

**DOI:** 10.1371/journal.pone.0059810

**Published:** 2013-03-28

**Authors:** Melania Melis, Maria Carla Aragoni, Massimiliano Arca, Tiziana Cabras, Claudia Caltagirone, Massimo Castagnola, Roberto Crnjar, Irene Messana, Beverly J. Tepper, Iole Tomassini Barbarossa

**Affiliations:** 1 Department of Biomedical Sciences, Section of Physiology, University of Cagliari, Monserrato, Italy; 2 Dipartimento di Scienze Chimiche e Geologiche, Università degli Studi di Cagliari, Monserrato, Italy; 3 Department of Life and Environment Sciences, Macrosection of Biomedicine, University of Cagliari, Monserrato, Italy; 4 Institute of Biochemistry and Clinical Biochemistry, Catholic University, Rome, Italy; 5 Department of Food Science, School of Environmental and Biological Sciences, Rutgers University, New Brunswick, New Jersey, United States of America; German Institute for Human Nutrition, Germany

## Abstract

The genetic predisposition to taste 6-n-propylthiouracil (PROP) varies among individuals and is associated with salivary levels of Ps-1 and II-2 peptides, belonging to the basic proline-rich protein family (bPRP). We evaluated the role of these proteins and free amino acids that selectively interact with the PROP molecule, in modulating bitter taste responsiveness. Subjects were classified by their PROP taster status based on ratings of perceived taste intensity for PROP and NaCl solutions. Quantitative and qualitative determinations of Ps-1 and II-2 proteins in unstimulated saliva were performed by HPLC-ESI-MS analysis. Subjects rated PROP bitterness after supplementation with Ps-1 and II-2, and two amino acids (L-Arg and L-Lys) whose interaction with PROP was demonstrated by ^1^H-NMR spectroscopy. ANOVA showed that salivary levels of II-2 and Ps-1 proteins were higher in unstimulated saliva of PROP super-tasters and medium tasters than in non-tasters. Supplementation of Ps-1 protein in individuals lacking it in saliva enhanced their PROP bitter taste responsiveness, and this effect was specific to the non-taster group.^1^H-NMR results showed that the interaction between PROP and L-Arg is stronger than that involving L-Lys, and taste experiments confirmed that oral supplementation with these two amino acids increased PROP bitterness intensity, more for L-Arg than for L-Lys. These data suggest that Ps-1 protein facilitates PROP bitter taste perception and identifies a role for free L-Arg and L-Lys in PROP tasting.

## Introduction

The ability to detect bitterness may have evolved to protect human beings from ingesting bitter-tasting toxins from plants and the environment. Humans possess an array of ∼25 bitter receptors that are capable sensing thousands of natural and synthetic compounds that impart bitter taste [1]–[4]. Some of these receptors are generalists, activated by many, chemically-diverse compounds (broadly tuned), whereas others are specialists, responding to only a single or a few compounds with closely-related structures [5].

Individuals vary in their perception of bitterness, and this variation is in part, genetically-determined. Genetic variability in taste sensitivity to thiourea derivatives, such as phenylthiocarbamide (PTC) and 6-n-propylthiouracil (PROP), is one of the most-studied human traits [6]. Both PROP and PTC contain a thiourea functional group (SC(NHR)_2_), which is responsible for their bitter taste [7]–[9]. The thiourea moiety is also a constituent of naturally-occurring glucosinolates that are present in bitter-tasting plants of the *Brassica* family. Studies have shown that taste responsiveness to PTC/PROP is associated with greater perception of bitterness from glucosinolate-containing [10] and other bitter vegetables and fruits [11] as well as decreased liking and intake of these foods [12]–[17]. Since PROP status is also associated with individual differences in fat perception and liking, energy intake and body weight, it has often been used as an oral marker for general food preferences and dietary behavior with subsequent links to body composition [11], [18]–[21]. Other taste receptor variants have been identified in humans that are important for bitter taste perception and liking [22]–[24]. However, these variants do not function as broad-based genetic markers of chemosensory responsiveness as has been attributed to PROP phenotype.

Individuals can be classified into three PROP taster categories: non-tasters, medium tasters, and PROP super-tasters based on suprathreshold measures at higher concentrations [18,19,21 25–31]. Non-tasters and PROP super-tasters illustrate the extremes of the phenotype with non-tasters showing little or no taste responsiveness to PROP, and PROP super-tasters experiencing intense bitter sensation from the compound. Medium tasters experience moderate bitterness sensation. Other reports suggest that PROP tasting may be a more continuous phenotype [18,19,27,28 32].

A growing literature in this field has focused on understanding and identifying the factors contributing to these large phenotypic differences in PROP bitter taste perception [32]–[35]. The ability to taste PROP is associated with haplotypes of the *TAS2R38* gene. Three amino acid substitutions (Pro49Ala, Ala262Val, and Val296Ile) in the sequence of this gene express variants of the receptor that bind the C = S moiety of the thiourea group [27], [32], [36]. Individuals homozygous for the AVI haplotype experience total taste blindness to PROP or a mild bitterness, whereas those homozygous or heterozygous for the PAV haplotype can taste PROP bitterness even at low concentrations. Other haplotypes (AAV, AAI, and PVI) have been observed rarely or are limited to specific populations [6]. Allelic diversity in *TAS2R38* accounts for the majority but not all of the phenotypic variation in PROP bitterness, thus implying the involvement of other factors [11], [28], [36], [37]. Indeed, family segregation, family-based linkage and genome-wide association studies suggest that other modifying genes may play a role in individual differences in PROP sensitivity [23], [38], [39]. Recent studies demonstrate that polymorphism rs2274333 (A/G) in the gene that codes for the salivary protein gustin (CA6) is also associated with PROP taster status in an ethnically homogeneous cohort [35], [40]. Specifically, the majority of PROP super-tasters also expressed the AA (active) form of gustin, whereas the majority of non-tasters expressed the GG (inactive) form of gustin. Gustin is thought to be a trophic factor for taste bud development and maintenance [41]. PROP phenotype is modulated by the apparent cooperation between *TAS2R38* and gustin polymorphisms, and the latter may explain why PROP super-tasters have a greater density of fungiform taste papillae which may contribute to their heightened oral chemosensory responsiveness.

As early as 1932, Fox [7] speculated that the inability to taste PTC/PROP was due to the presence of a product (perhaps a protein) in the saliva of non-tasters that precipitated the PROP molecule and interfered with its perception. This hypothesis received partial and indirect support from experiments indicating that the stimulating capability of a taste stimulus depends on its solubility [42]. However, other evidence suggests that PTC non-taster condition is unlikely to depend on the lack a salivary component that permits PTC to be tested [43]–[45]. Our laboratory has been studying the involvement of salivary proteins in PROP tasting [46]. We recently showed that PROP status is associated with basal levels of two salivary peptides belonging to the basic proline-rich protein family (bPRP), namely Ps-1 and II-2, which are both encoded by the *PRB1* gene [47]. In particular, we demonstrated that greater PROP bitterness was related to higher concentrations of the Ps-1 protein and II-2 peptide compared with lower PROP bitterness. The functional significance of these two proteins in the saliva of PROP super-tasters is currently unknown. The best-known function of PRPs is their ability to precipitate and neutralize the negative biological effects of tannins during the development of oral astringency [48]–[53]. Establishing another role for PRPs in PROP bitterness perception would extend our understanding of their biological functions and demonstrate their importance within a broader nutritional context.

**Figure 1:**
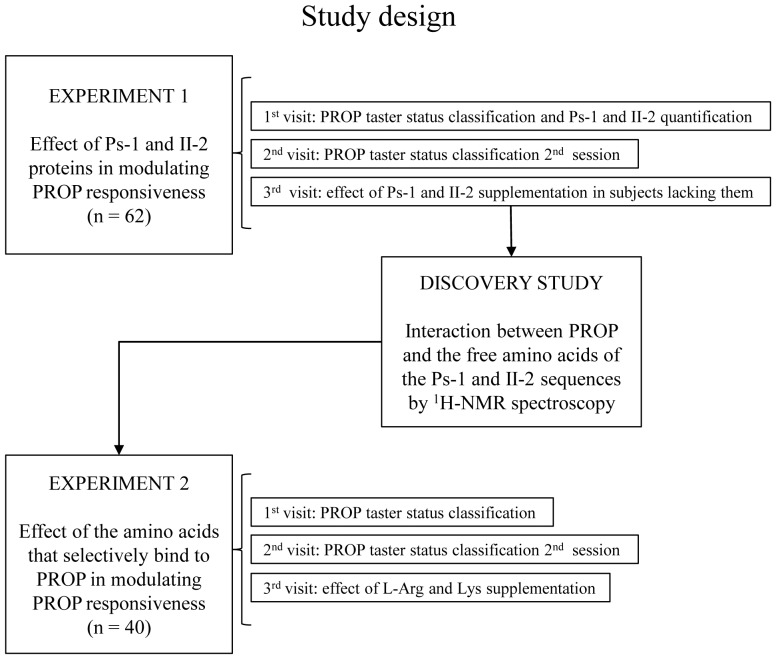
Graphic diagram representing the study design.

The purpose of this work was to gain insight into the physiological mechanisms by which Ps-1 and II-2 facilitate the perception of PROP bitterness in subjects classified by PROP phenotype. We administered Ps-1 and II-2 to individuals who lacked these proteins to determine if oral supplementation would lead to greater bitterness perception from PROP. ^1^H-NMR spectroscopy was used to chemically probe the interaction between PROP and the free amino acids present in the Ps-1 and II-2 sequences. This experiment identified two amino acids (L-arginine and L-lysine) involved in the local binding of these peptides to the PROP molecule. We then administered L-arginine and L-lysine to subjects to determine if oral supplementation with these amino acids enhanced the bitterness of PROP. The overall design of the study is depicted in Figure 1.

## Materials and Methods

### Ethics Statement

Ethics approval was obtained from the Ethical Committee of the University Hospital of Cagliari, and the study has therefore been performed in accordance with the ethical standards laid down in the 1964 Declaration of Helsinki. All subjects reviewed and signed an informed consent form.

### Subjects

One hundred and two non-smoking subjects were recruited through public advertisements at the University of Cagliari. All were healthy white men (n = 35) and women (n = 67), their average age being 27.6 y±1.2 y and with a body mass index (BMI) ranging from 18.6 to 25.3 kg/m^2^. They had no variation in body weight larger than 5 kg recorded over the previous 3 months, and were not following a prescribed diet or taking medications that might interfere with taste function. Subjects neither had food allergies, nor scored high on eating behaviour scales (assessed by the Three-Factor Eating Questionnaire) [54]. In order to rule out any gustatory impairment, thresholds for sweet, sour, salty, and bitter tastes were determined for all participants. None of the participants was ageusic. At the beginning of the protocol, before signing an informed consent form, each subject was verbally instructed about the procedure and the aim of the study.

### Experimental Procedures

All subjects were requested to abstain from eating, drinking and using oral care products or chewing gums for at least 8 h prior to taste tests that were completed in three visits in both experiments (1 and 2). They had to be in the test room 15 min before the beginning of the trials (at 9.30 AM) in order to adapt to the environmental conditions (23–24°C; 40–50% relative humidity) which were kept constant throughout the experimental sessions. In the first visit, before starting taste assessments, 1 mL sample of whole unstimulated saliva was collected for the Ps-1 and II-2 quantitative determination by HPLC-ESI-IT-MS analysis as described below. In women, the taste assessments and saliva collection were done on the sixth day of the menstrual cycle to minimize taste sensitivity changes and value fluctuations due to the estrogen phase [55], [56].

For all taste assessments, the solutions were prepared the day before each session and stored in the refrigerator until 1 h before testing. Stimuli were presented at room temperature. The taste intensity rating for each solution was recorded by using the Labeled Magnitude Scale (LMS) [57] in which each subject placed a mark on the scale corresponding to his/her perception of the stimulus. The LMS scale gives subjects the freedom to rate the intensity of a stimulus relative to the “strongest imaginable” oral stimulus they have ever experienced in their life.

### PROP Screening and Taster Status Classification

Subjects were assessed for PROP taster status using the 3-solution test [30], [58]. Taste intensity ratings were collected for three suprathreshold PROP (Sigma-Aldrich, Milan, Italy) (0.032, 0.32, and 3.2 mM) and sodium chloride (NaCl, Sigma-Aldrich, Milan, Italy) (0.01, 0.1, 1.0 M) solutions dissolved in spring water. NaCl was used as standard as previously done in other studies [12], [25], [30]. Subjects were classified for PROP taster status in two visits that were separated by a 1-month period. The presentation order of the two taste stimuli (10 mL) (PROP or NaCl) was reversed in the two visits, and concentrations within each solution type were tasted in a random order. An oral rinsing with spring water followed each stimulation. The interstimulus interval was set at 60 s.

The mean of ratings in the two replicates was calculated and perceived taste intensity functions for PROP and NaCl for each subject were generated from the results [12], [30]. When intensities of PROP ratings increased more steeply across concentrations than those of NaCl ratings, the subject was classified as a “PROP super-taster” (n = 36). On the contrary, when the NaCl ratings increased more steeply than did the PROP ratings, the subject was classified as a non-taster (n = 35). When the PROP ratings overlapped with the NaCl ratings, the subject was classified as a medium taster (n = 31). ANOVA was used to document the presence of the three taster groups (see Table S1).

According to the study design (see Figure 1), subjects were divided into two pools. The first subject pool (n = 62) was composed of 24 PROP super-tasters; 17 medium tasters and 21 non-tasters who rated the bitterness of PROP after oral supplementation with PS-1 or II-2. The second pool (n = 40) was composed of 12 PROP super-tasters; 14 medium tasters and 14 non-tasters who rated the bitterness of PROP after oral supplementation with L-Arg and L-Lys.

### Ps-1 and II-2 Salivary Protein Analyses

#### Saliva collection and treatment

A sample (1 mL) of whole unstimulated saliva was collected from sixty-two subjects with a soft plastic aspirator as it flowed into the anterior floor of the mouth for less than 1 min, and then transferred to a plastic tube. Each sample was immediately mixed with an equal volume of aqueous trifluoroacetic acid (0.2%) in an ice bath, in order to preserve and stabilize the sample by inhibiting salivary proteases. The solution was then centrifuged at 8000 g, and kept at 4°C for 15 min. The acidic supernatant was separated from the precipitate and then immediately stored at −80°C until the HPLC-ESI-IT-MS analysis.

#### HPLC-ESI-IT-MS analysis

Ps-1 and II-2 proteins were identified and quantified in each of the sixty-two samples, by HPLC-ESI-IT-MS according to Cabras et al. [46]. 100 µL of the acidic soluble fraction corresponding to 50 µL of whole unstimulated saliva was used. Identification was based on the chromatographic behavior and comparison of the experimental mass values with the theoretical ones reported in the Swiss-Prot Data Bank (http://us.expasy.org/tools). The quantification of Ps-1 and II-2 proteins was based on the area of the RP-HPLC-ESI-MS extracted ion current (XIC) peaks. The XIC analysis reveals the peak associated with the protein of interest by searching along the total ion current chromatographic profile of the specific multi-charged ions generated at the source by the protein. The area of the ion current peak is proportional to concentration, and under constant analytical conditions it may be used to perform relative quantification of the same analyte in different samples [59], [60].

#### Ps-1 and II-2 proteins purification

To purify Ps-1 and II-2 proteins, a volume of 35 mL of whole saliva was collected from a single healthy female volunteer in our laboratory after she signed an informed consent. The whole saliva was treated as previously described and the volume of the acidic soluble fraction reduced by lyophilization to ca 2 mL was stored at −80°C until purification.

The concentrated acidic soluble fraction of 35 mL of whole saliva was submitted to gel-filtration on a Sephadex-G 75 column (44×3 cm) equilibrated with 20 mM sodium acetate buffer, pH 4.8, at a flow rate of 0.35 µL/min. Fractions of 1 mL were collected and checked at 214 and 276 nm. Six pools were collected on the basis of the elution profile. Each pool was concentrated by lyophilization and then dialyzed against ultra-pure deionized water. HPLC-ESI-MS revealed that pool 2 contained almost pure Ps-1 and pool 5 almost pure II-2. The XIC peak area/mL was measured for both proteins.

### PROP Bitterness Assessments after Supplementation with Ps-1 and II-2 Proteins

The concentration of each bPRP added to PROP solution (3.2 mM) corresponded to the average amount of the protein determined in1 mL of the PROP super-taster unstimulated saliva, as established on the basis of the XIC peak area (Ps-1∶1.33×10^9^ and II-2∶1.55×10^9^ a.u.).

In a third visit, the effect of the Ps-1 or II-2 supplementation on PROP bitterness was assessed in subjects of the first pool who were lacking in Ps-1 (n = 20) or II-2 (n = 7), respectively. Briefly, all rinsed their mouth with spring water before starting. Each subject was presented, in a random order, with 2 cups (4 mL samples) one containing only PROP and the other PROP supplemented with Ps-1 or II-2. They were instructed to swish the entire contents of one cup in their mouth for 10 s and then to spit it out. Each stimulation was followed by oral rinsing with spring water. The interstimulus interval was set at 5 min. After 1 h each subject was presented with two other cups (controls) one containing only the Ps-1 protein and the other only the II-2 peptide at the same concentrations previously used. The intensity rating for each solution was collected by having the subject place a mark on the LMS scale corresponding to his/her perception of the stimulus.

### ^1^H-NMR Spectroscopy-PROP/Amino Acid Binding

The interaction between PROP and the free form of amino acids present in the Ps-1 and II-2 sequences was investigated by ^1^H-NMR spectroscopy. This technique is a powerful analytical tool capable of identifying and quantifying a large number of compounds having hydrogen atoms, and it has been already employed in evaluating the interaction of proteins and/or specific amino-acid sequences with tannins and polyphenols [61]. A proton involved in the interaction with an external molecule experiences a modification in its chemical surrounding that implies a field-shift and a change of the corresponding ^1^H-NMR signal. Thus, when such an interaction occurs, a variation of the chemical shift of the protons belonging to the amino acids of the protein directly involved in the local binding is expected. We individually recorded the ^1^H-NMR spectra of all the amino acids present in the Ps-1 and II-2 sequences before and after the addition of an equimolar amount of PROP.

All experiments were recorded at 300 K using a Varian Inova 500 MHz FT-NMR system.

Spectra were processed and displayed using the MestReNova program. The experiments were performed by preparing 0.5 mL of a 5 mM solution of each amino acid in D_2_O and then recording the corresponding spectrum. Afterwards, an equimolar amount of a PROP solution in D_2_O was added to each amino acid solution and the ^1^H-NMR spectrum recorded. Chemical shifts for ^1^H NMR are reported in parts per million (ppm), calibrated to the residual solvent peak set, with coupling constants reported in Hertz (Hz).The following abbreviations are used for spin multiplicity: s = singlet, d = doublet, t = triplet, m = multiplet, dd = doublet of doublets. The ^1^H-NMR chemical shift change for the PROP ring proton in the absence and in the presence of each amino acid was determined in terms of Δ = (|(δ’−δ_0_)|/δ_0_)·100, which represents the absolute value of the difference between the ^1^H-NMR signal (ppm) of the PROP ring proton in the absence (δ_0_) and in the presence (δ’) of the amino acid, normalized for δ_0_ and expressed as a percentage.

### PROP Bitterness Assessments after Supplementation with L-Arginine and L-Lysine

On the basis of the results obtained in the ^1^H-NMR spectroscopy binding study, the effect of L-Arg and L-Lys supplementation on PROP (3.2 mM) bitterness was assessed in a third visit of second pool subjects. Each subject was presented, in a random order, with 3 cups (4 mL samples): one containing only PROP, one with PROP supplemented with L-Arg, and one with PROP supplemented with L-Lys. After 1 h, each subject was presented with two more cups, one containing only L-Arg and the other containing only L-Lys. The procedure for collecting the taste intensity ratings was the same as the one described for the supplementation of Ps-1 and II-2 proteins. Concentrations of L-Arg (prepared from the hydrochloride salt, Sigma-Aldrich, Milan, Italy) and L-Lys (Sigma-Aldrich, Milan, Italy) were 3.2 mM.

### Statistical Analyses

The Kruskal-Wallis test was used to compare the concentrations of the Ps-1 protein and II-2 peptide in unstimulated saliva of PROP super-tasters, medium tasters and non-tasters, and to evaluate gender differences. The Fisher exact test was used to compare the percentage of subjects lacking Ps-1 or II-2 across PROP taster groups. Repeated measures ANOVA was used to analyse the effects of supplementation with the two proteins (Ps-1 and II-2) or the two amino acids (L-Arg and L-Lys) on PROP bitterness intensity. Post-hoc comparisons were conducted with the Newman-Keuls test. Statistical analyses were conducted using STATISTICA for WINDOWS (version 7; StatSoft Inc, Tulsa, OK, USA). *P* values<0.05 were considered significant.

### Nomenclature

When genes and the encoded proteins share the same acronym, the name of the gene is identified in italics, while its corresponding encoded protein by plain text.

## Results

### Ps-1 or II-2 Oral Supplementation

**Figure 2:**
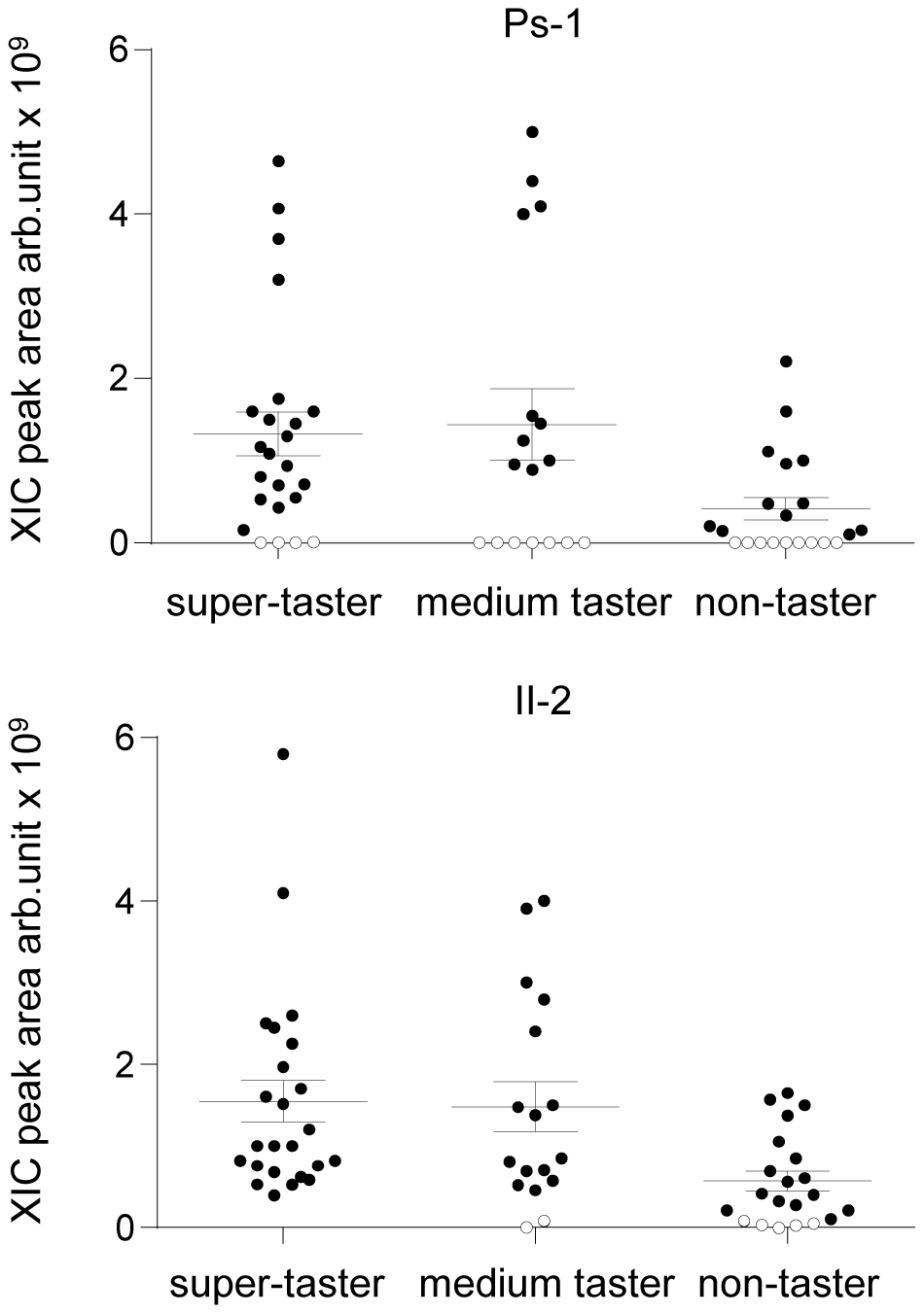
Relative concentrations of Ps-1 and II-2 in the PROP taster groups in unstimulated (resting) saliva. Distribution of the XIC peak areas of Ps-1 and II-2 and mean values ± SEM for each taster group are reported. Ps-1 mean values were lower in non-tasters than in PROP super-tasters and those of II-2 were lower in non-tasters relative to the other groups (Ps-1: *p* = 0.0216; II-2: *p*≤0.004; Kruskal-Wallis test). Out of 62 subjects, n = 21 non-tasters, n = 17 medium tasters and n = 24 PROP super-tasters. Subjects lacking Ps-1 (n = 20) or II-2 (n = 7) in their saliva are identified by white circles.

Figure 2 shows the distributions of the relative concentrations of the Ps-1 protein and II-2 peptide determined by HPLC-ESI-IT-MS analysis in unstimulated saliva of PROP super-tasters, medium tasters and non-tasters. The Kruskal-Wallis test showed that mean values of the extract ion current (XIC) peak areas of Ps-1 and II-2 depend on PROP taster status (Ps-1: *H*[_2,62_] = 7.573, *p* = 0.02 and II-2: *H*[_2,62_] = 14.958, *p* = 0.0006). Pairwise comparisons showed that Ps-1 concentration was significantly lower in saliva of non-tasters than in PROP super-tasters (Ps-1: *p* = 0.0216), and that of II-2 was significantly lower in saliva of non-tasters than in PROP super-tasters and medium tasters (*p*≤0.004). The figure also shows that several individuals were lacking these proteins. The Ps-1 protein was undetected in a total of 20 subjects, while the II-2 peptide was undetected in only 7 subjects. Additionally, the percentage of non-tasters lacking Ps-1 (43%) was higher from that of PROP super-tasters (17%) although at the limit of statistical significance (*p* = 0.053), while the percentage of medium tasters (41%) was not different from that of the other taster groups (p≥0.08). The same pattern was observed for II-2. The percentage of non-tasters lacking II-2 (24%) was statistically different from PROP super-tasters (all had II-2) (*p* = 0.017), while medium tasters (12%) were not different from the other taster groups (p≥0.08). No changes in the salivary proteome were related to gender (Ps-1: *H*[_1,62_] = 0.148, *p* = 0.700 and II-2: *H*[_2,62_] = 0.144, *p* = 0.704).

**Figure 3:**
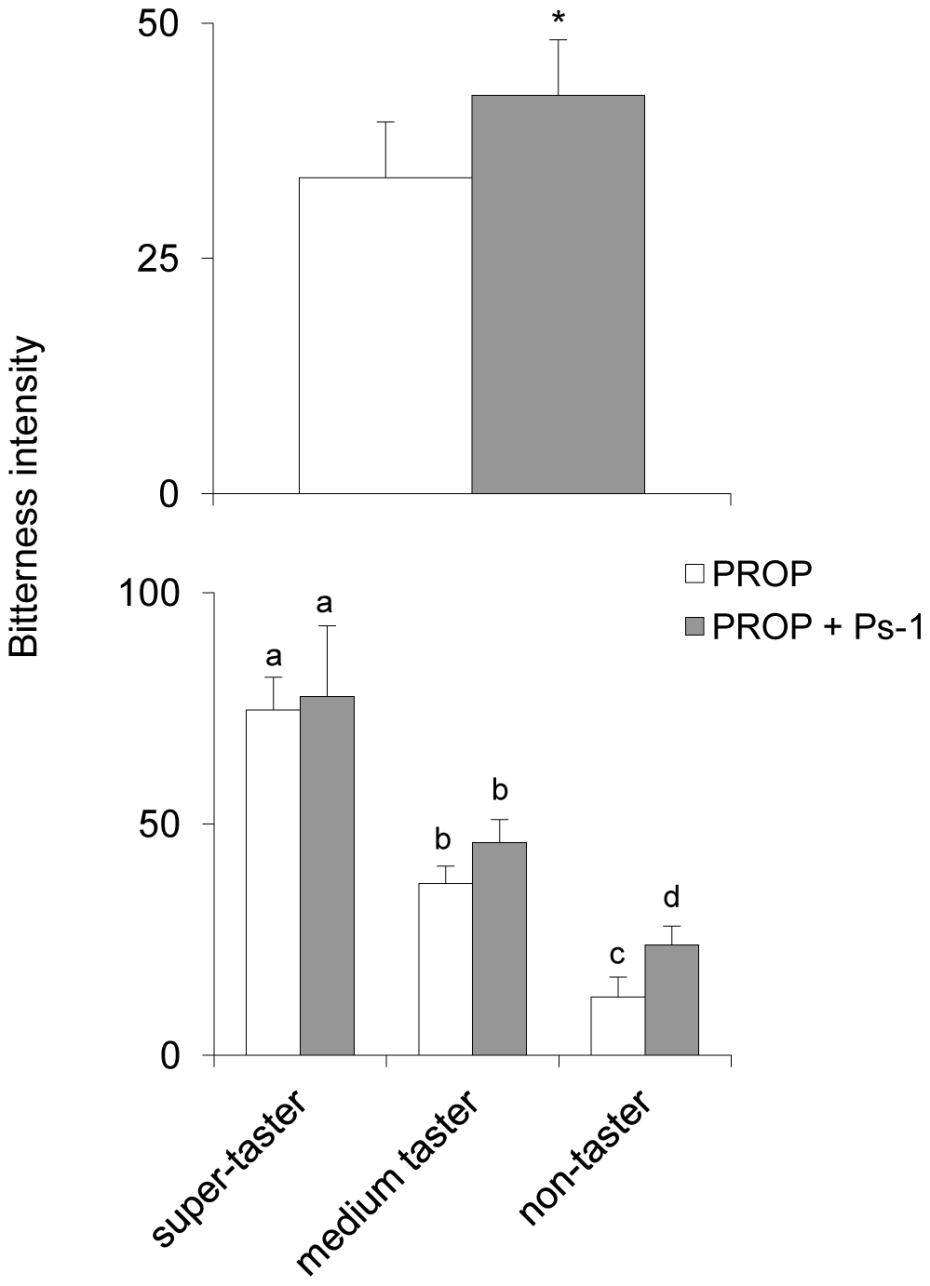
Effect of the Ps-1 protein on PROP bitterness intensity. Mean (± SEM) bitterness intensity evoked by PROP and PROP+Ps-1 solutions (upper graph) in 20 subjects lacking Ps-1. The same data are shown in the lower graph for each taster group (n = 9 non-tasters; n = 7 medium tasters; n = 4 PROP super-tasters). The solution containing only Ps-1 (control) is not shown as it did not evoke any taste perception. * = significant difference (*F*[_1,17_] = 7.2273, *p* = 0.0155; repeated measures ANOVA). Different letters indicate significant differences (*p*≤0.0012; Newman-Keuls test subsequent to repeated measures ANOVA).

Repeated measures ANOVA revealed that PROP bitterness intensity of individuals lacking Ps-1 protein significantly increased after supplementation of this protein (*F*[_1,17_] = 7.2273; *p* = 0.0155) (Figure 3). Post-hoc comparisons showed that Ps-1 supplementation significantly increased the PROP bitterness intensity in non-tasters (*p* = 0.0367; Newman-Keuls test), but not in the other two taster groups (*p*>0.05) (Figure 2, lower graph). The solution containing only protein did not evoke any taste perception (data not shown). The supplementation of II-2 peptide did not produce the same effect (*p*>0.05) (data not shown).

### ^1^H-NMR Spectroscopy – PROP Binding

**Figure 4:**
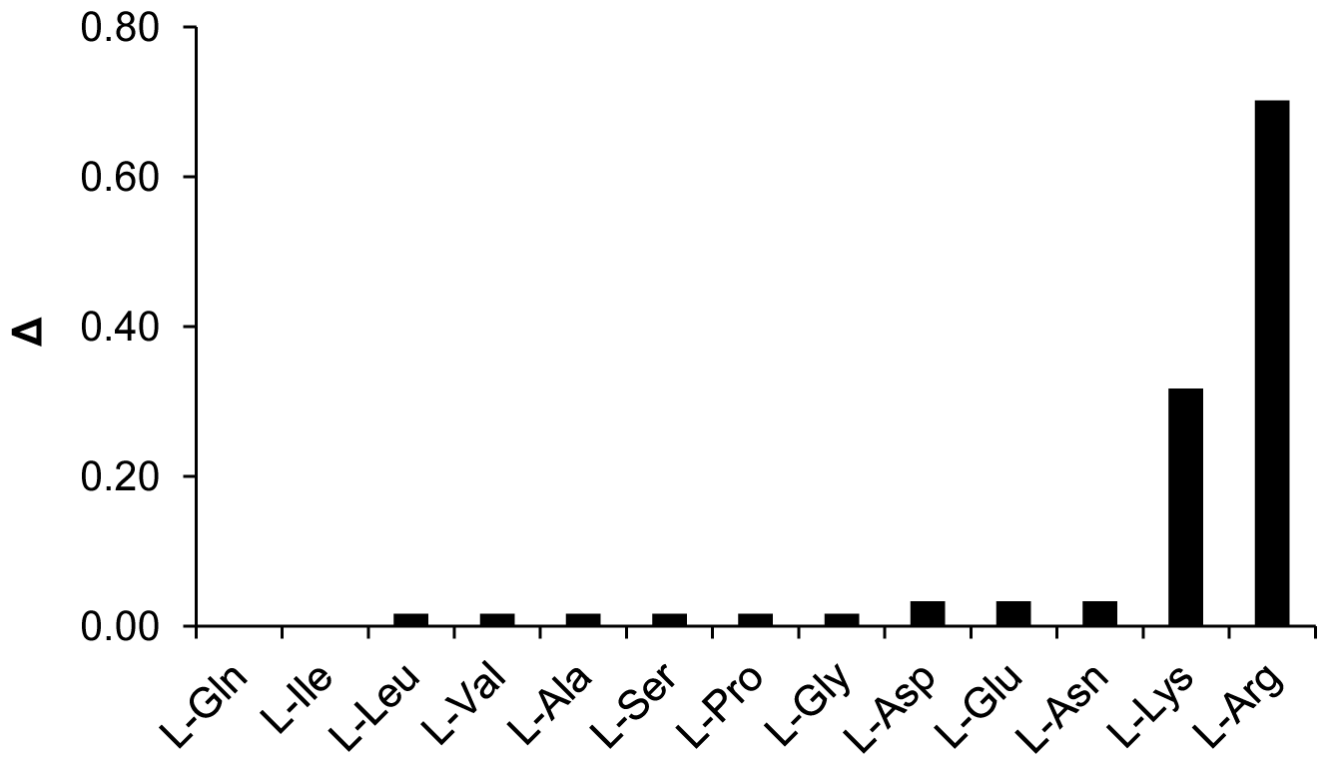
PROP ring proton ^1^H-NMR chemical shift variation upon amino acids addition reported as Δ. Δ = (|(δ’−δ_0_)|/δ_0_)·100 represents the absolute value of the difference between the ^1^H-NMR signal (ppm) of the PROP ring proton in the absence (δ_0_) and in the presence (δ’) of the amino acid of the Ps-1 and II-2 sequences, normalized for δ_0_ and expressed as a percentage. For each amino acid, two spectra were recorded in 0.5 mL of 5 mM D_2_O solution before and after the addition of an equimolar amount of PROP.

^1^H-NMR spectroscopy allowed us to determine the proton assignments for all analyzed amino acids before and after the addition of an equimolar amount of PROP (Table 1). It is interesting to note that after PROP addition, a chemical shift variation occurs only in the protons belonging to L-Arg and L-Lys, while the ^1^H-NMR signals for the other amino acids remained unchanged. Accordingly, the ring proton in the PROP molecule undergoes a chemical shift in the 1H-NMR signal in the presence of L-Arg and L-Lys only (Figure 4).

**Table 1:** ^1^H -NMR assignments (ppm) for the amino acids of Ps-1 and II-2 sequences, and PROP^a^.

←	←^1^H-Cα	←^1^H-Cβ	←^1^H-Cγ	←^1^H-Cδ	←^1^H-Cε	←^1^H-Cβ’
←Ala	←3.83 (q)	←1.53 (d)	←	←	←	←
←Arg	←3.26t	←1.66–1.73 (m)	←1.66–1.73 (m)	←3.38t	←	←
←	←(3.28t)^b^	←(1.67–1.75m)^b^	←(1.67–1.75m)^b^	←(3.6br)^b^	←	←
←Asn	←4.06 (q)	←2.95 (dd)	←	←	←	←
←Asp	←4.10 (t)	←3.03 (dd)	←	←	←	←
←Gly	←3.61 (s)	←	←	←	←	←
←Glu	←3.86 (t)	←2.15–2.26 (m)	←2.59–2.63 (m)	←	←	←
←Gln	←3.82 (t)	←2.19 (q)	←2.48–2.55 (m)	←	←	←
←Ile	←3.72 (d)	←2.00–2.05 (m)	←1.32.–1.55(m)	←1.00 (t)	←	←1.06 (d)
←Leu	←3.78 (t)	←1.71–1.82 (m)	←1.71–1.82 (m)	←1.01 (t)	←	←
←Lys	←3.61t	←1.76–1.85 (m)	←1.40–1.60 (m)	←1.74t	←3.06 (t)	←
←	←(3.8br)^b^	←(1.92–2.00 m, br)^b^	←(1.40–1.60 m, br)^b^	←(1.77t)^b^	←(3.07t)^b^	←
←Pro	←4.18 (t)	←2.38–2.53 (m)	←2.04–2.15 (m)	←3.37–3.50 (m)	←	←
←Ser	←3.94–4.06(m)	←3.89 (t)	←	←	←	←
←Val	←3.66 (d)	←2.29–2.36 (m)	←1.07 (dd)	←	←	←

a PROP assignments (ppm): 1.00t [**CH_3_**(CH_2_CH_2_)]; 1.70q [(CH_3_)**CH_2_**(CH_2_)]; 2.52t [(CH_3_CH_2_)**CH_2_**]; 5.985s H(**CH**).

b Chemical shifts upon PROP addition are reported in parentheses only when changed.

NMR signal descriptions: s (singlet); d (doublet); t (triplet); q (quadruplet); m (multiplet); br (broad signal); dd (doublet of doublets).

### L-Arg or L-Lys Oral Supplementation

**Figure 5:**
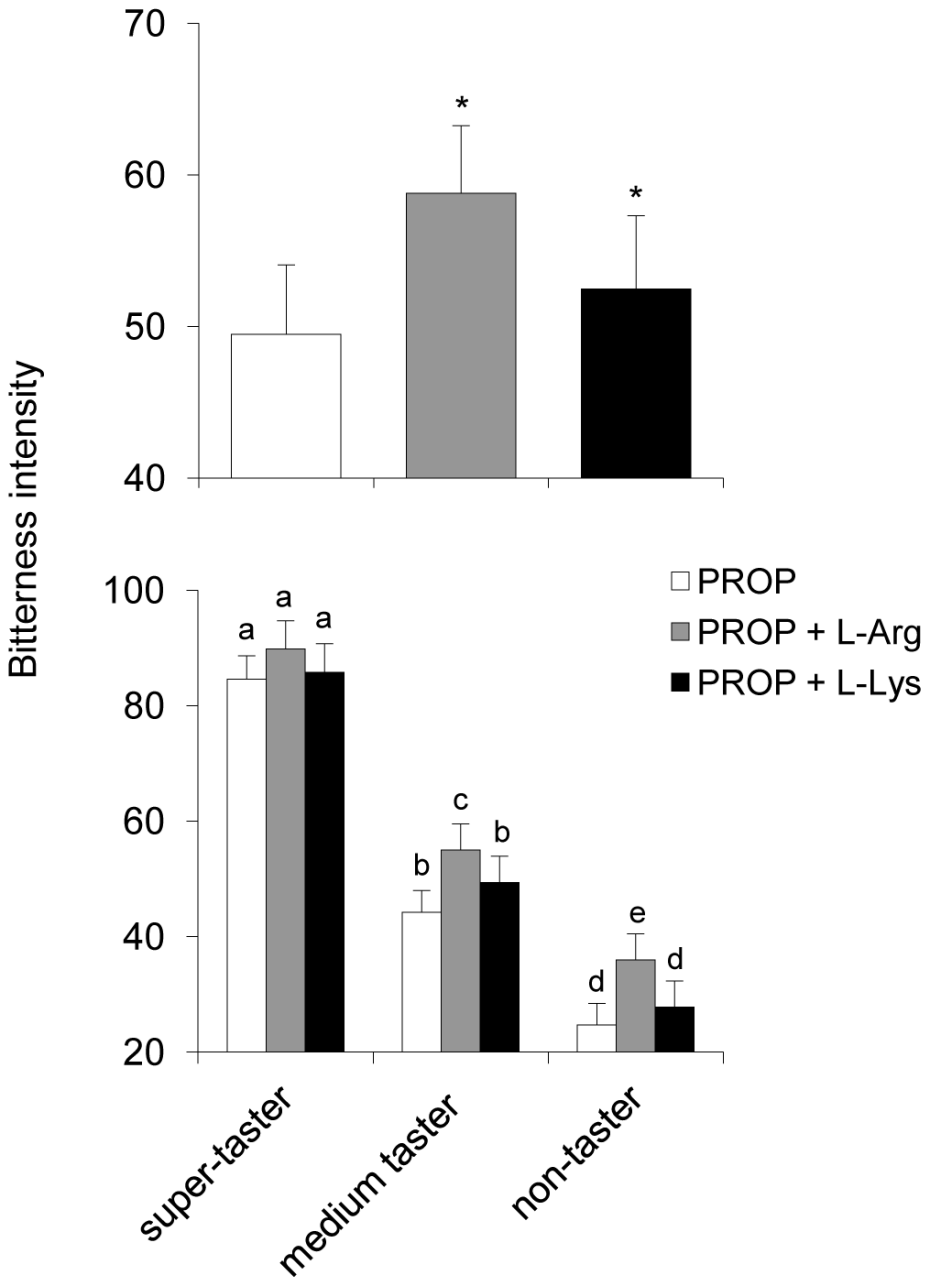
Effect of L-Arg or L-Lys supplementation on PROP bitterness intensity. Mean values ± SEM of bitterness intensity evoked by PROP, PROP+L-Arg and PROP+L-Lys solutions in a group of 40 subjects (upper graph). The same data are shown in the lower graph for each taster group (14 non-tasters; 14 medium tasters; 12 PROP super-tasters). Control solutions containing only L-Arg or L-Lys are not shown as they did not evoke any taste perception. * = significantly different from PROP (PROP +L-Arg: *F*[_1,37_] = 27.124, *p* = 0.00001and PROP+L-Lys: *F*[_1,37_] = 5.949, *p* = 0.0196; repeated measures ANOVA). Different letters indicate significant differences (*p*≤0.0012; Newman-Keuls test subsequent to repeated measures ANOVA).

The effect of L-Arg or L-Lys supplementation on PROP bitterness intensity in 40 subjects of experiment 2 is shown in Figure 5. Repeated measures ANOVA revealed that PROP bitterness intensity significantly increased after supplementation with either of the two amino acids (L-Arg: *F*[_1, 7_] = 27.124, *p* = 0.00001 and L-Lys: *F*[_1,37_] = 5.949, *p* = 0.0196) (upper graph). Post-hoc comparisons showed that L-Arg supplementation significantly increased the PROP bitterness intensity in non-tasters and medium tasters (*p*≤0.0012; Newman-Keuls test), but not in PROP super-tasters (*p*>0.05). Instead, post hoc comparison showed no significant differences in the case of L-Lys supplementation (*p*>0.05). The solutions containing only L-Arg or L-Lys did not evoke any taste perception (data not shown).

## Discussion

The best-known function of salivary PRPs is their ability to bind and precipitate tannins in the oral cavity during astringency perception [48]–[53]. The present data provide new insights into the roles of Ps-1, II-2 and their constituent amino acids in PROP taste perception. First, in agreement with our previous findings [46], we showed that non-tasters had the lowest concentration of Ps-1 and II- 2 proteins in their saliva compared to PROP super-tasters who had the highest concentrations. In addition, we found that many non-tasters and medium tasters lack the two proteins in their saliva, while all or almost all PROP super-tasters have them. The lack of these two proteins in a large number of medium tasters (50% for Ps-1) is consistent with the moderate PROP responsiveness of individuals in this group.

Importantly, oral supplementation with Ps-1 in individuals lacking this protein in saliva enhanced their PROP bitter taste responsiveness, and this effect was most potent in non-tasters (Fig. 3). Since relatively few subjects (∼11%) lacked salivary II-2, we could not test the effects of supplementation with this peptide on PROP bitterness.

To better understand the mechanism by which the Ps-1 protein increases PROP bitterness, we investigated the interaction between PROP and the free form of the constituent amino acids of the Ps-1 and II-2 sequences by ^1^H-NMR spectroscopy. Our results indicate that only L-Arg and L-Lys, among all the amino acids in the sequences of the two proteins, interact with the PROP molecule, and the interaction between PROP and L-Arg is stronger than that involving L-Lys. Since L-Lys and L-Arg are the only amino acids displaying terminal amino-groups among those we studied, the ^1^H-NMR measurements suggest that the interaction could involve these terminal groups and the carbonyl/thiocarbonyl groups of the PROP heterocycle.

Our psychophysical data strongly support the ^1^H-NMR results, showing that L-Arg enhances the bitterness intensity of PROP more than L-Lys. Moreover, similar to our observations for Ps-1 supplementation, the effect of L-Arg on PROP bitterness intensity was restricted to non-tasters and medium tasters, and not PROP super-tasters. No changes in bitterness perception were related to PROP status in the case of L-Lys supplementation. It is worth noting that the bitter taste ratings of these amino acids are very low at high concentrations [62], and we found that L-Arg and L-Lys were tasteless at the concentrations used in this study.

The present findings may have implications for understanding the structural features and binding properties of the TAS2R38 receptor. According to recent studies, the predicted binding sites and binding affinity for PROP and PTC to the TAS2R38 receptor vary across *TAS2R38* haplotypes [32], [33], [63]. Specifically, the hydrogen bond interaction between the transmembrane domain (TM) 3 and amino acid 262 in TM6 is involved in the interhelical network that permits the activation of the G protein-coupled receptor (GPCR) in the PAV haplotype but not in the AVI haplotype. Furthermore, the H bond between the PROP molecule and residue 262 in the PAV haplotype is involved in bitter tasting. Based on these considerations and our own findings, we can speculate that the Ps-1 protein could be involved in orienting the PROP molecule within the binding pocket to optimize its binding when the receptor has the PAV form. The fact that supplementation with Ps-1 in non-tasters increased PROP bitterness suggests that, even without an optimal binding pocket, the Ps-1 protein can help the PROP molecule twist and turn in order to facilitate its binding with the receptor in the AVI form. Considering that we observed a similar effect with L-Arg supplementation, and that this amino acid is highly represented in the protein sequence (7 occurrences), we suppose that the permissive function of the Ps-1 protein on PROP tasting might be carried out via L-Arg. In order to confirm this hypothesis, future studies will analyze the three-dimensional structure of the protein in order to verify whether the spatial positions of the arginine residues are suitable for binding the stimulus.

In conclusion, this work further elucidates the role of the salivary proteome in PROP taste perception and highlights the importance of the Ps-1 protein and its constituent amino acids (L-Arg and L-Lys) in receptor binding and activation. Future studies will have to determine if the effects of Ps-1 on bitter taste enhancement are unique to PROP tasting or whether Ps-1 has broader effects on bitter taste function.

## Supporting Information

Table S1 **Ratings of perceived taste intensity in response to three concentrations of PROP and NaCl in the taster groups.**
(DOC)
