# Supplementary material for: Marked Increase in PROP Taste Responsiveness Following Oral Supplementation with Selected Salivary Proteins or Their Related Free Amino Acids
Source: PLoS One. 2013 Mar 28;8(3):e59810. doi: 10.1371/journal.pone.0059810 (PMC3610910; doi:10.1371/journal.pone.0059810)
Supplement: Table S1 — Ratings of perceived taste intensity in response to three concentrations of PROP and NaCl in the taster groups. (DOC) [file pone.0059810.s001.doc]

**Table S1.** Ratings of perceived taste intensity in response to three concentrations of PROP and NaCl in the taster groups.

|  | super-tasters  (*n*=36) | medium tasters  (*n*=31) | non-tasters  (*n*=35) |
| --- | --- | --- | --- |
| PROP |  |  |  |
| 0.032 mM | 6.83 ± 0.93 | 3.91 ± 1.37 | 1.77 ± 0.50 |
| 0.32 mM | 38.51 ± 1.76* | 22.80 ± 1.60 | 5.80 ± 0.76* |
| 3.2 mM | 80.88 ± 2.83* | 49.81 ± 3.28 | 27.72 ± 2.79* |
|  |  |  |  |
| NaCl |  |  |  |
| 0.01 M | 1.63 ± 0.43 | 2.88 ± 1.10 | 7.15 ± 1.37 |
| 0.1 M | 16.17 ± 1.09* | 21.21 ± 2.07 | 34.87 ± 2.86* |
| 1 M | 40.05 ± 1.99* | 54.72 ± 3.37 | 65.01 ± 3.13* |

Values are means ± SEM. *n* = 102.Three-way ANOVA was used to compare PROP intensity ratings with NaCl intensity ratings across groups (*F*_[4,594]_ = 37.166; *p*<0.00001).

* = significant difference between PROP and the corresponding NaCl concentration (*p*<0.00001; Newman-Keuls test).
